# Virus-induced endothelial senescence as a cause and driving factor for ME/CFS and long COVID: mediated by a dysfunctional immune system

**DOI:** 10.1038/s41419-025-08162-2

**Published:** 2026-01-09

**Authors:** Massimo Nunes, Loren Kell, Anouk Slaghekke, Rob CI Wüst, Burtram C. Fielding, Douglas B. Kell, Etheresia Pretorius

**Affiliations:** 1 Department of Physiological Sciences, Faculty of Science, Stellenbosch University, Stellenbosch, South Africa; 2 Botnar Institute for Musculoskeletal Sciences, Nuffield Department of Orthopaedics, Rheumatology and Musculoskeletal Sciences (NDORMS), University of Oxford, Oxford, UK; 3 Department of Human Movement Sciences, Amsterdam Movement Sciences, Vrije Universiteit Amsterdam, Amsterdam, Netherlands; 4 Department of Microbiology, Faculty of Science, Stellenbosch University, Stellenbosch, South Africa; 5 Department of Biochemistry, Cell and Systems Biology, Institute of Systems, Molecular and Integrative Biology, Faculty of Health and Life Sciences, University of Liverpool, Liverpool, UK; 6 The Novo Nordisk Foundation Centre for Biosustainability, Building 220, Chemitorvet 200, Technical University of Denmark, Kongens Lyngby, Denmark

**Keywords:** Diagnostic markers, Thrombosis

## Abstract

Myalgic encephalomyelitis/chronic fatigue syndrome (ME/CFS) and long COVID are two post-viral diseases, which share many common symptoms and pathophysiological alterations. Yet a mechanistic explanation of disease induction and maintenance is lacking. This hinders the discovery and implementation of biomarkers and treatment options, and ultimately the establishment of effective clinical resolution. Here, we propose that acute viral infection results in (in)direct endothelial dysfunction and senescence, which at the blood-brain barrier, cerebral arteries, gastrointestinal tract, and skeletal muscle can explain symptoms. The endothelial senescence-associated secretory phenotype (SASP) is proinflammatory, pro-oxidative, procoagulant, primed for vasoconstriction, and characterized by impaired regulation of tissue repair, but also leads to dysregulated inflammatory processes. Immune abnormalities in ME/CFS and long COVID can account for the persistence of endothelial senescence long past the acute infection by preventing their clearance, thereby providing a mechanism for the chronic nature of ME/CFS and long COVID. The systemic and tissue-specific effects of endothelial senescence can thus explain the multisystem involvement in and subtypes of ME/CFS and long COVID, including dysregulated blood flow and perfusion deficits. This can occur in all tissues, but especially the brain as evidenced by findings of reduced cerebral blood flow and impaired perfusion of various brain regions, post-exertional malaise (PEM), gastrointestinal disturbances, and fatigue. Paramount to this theory is the affected endothelium, and the bidirectional sustainment of immune abnormalities and endothelial senescence. The recognition of endothelial cell dysfunction and senescence as a core element in the aetiology of both ME/CFS and Long COVID should aid in the establishment of effective biomarkers and treatment regimens.

## Facts

- Evidence of endothelial dysfunction and perfusion deficits in ME/CFS and Long COVID suggests a central role for vascular abnormalities in pathology and symptom manifestation, bringing into question the long-term effects of acute viral infections on the vasculature.
- Several of the viruses implicated, including SARS-CoV-2, influenza A, enteroviruses, and herpesviruses, can directly infect endothelial cells, and SARS-CoV-2 and influenza A have even been shown to induce endothelial senescence.
- The senescence-associated secretory phenotype of endothelial cells can lead to enhanced vasoconstriction and blood flow abnormalities, immune dysregulation, impaired tissue repair, elevations in oxidative stress, and coagulation abnormalities, and has the potential to account for various symptoms observed in ME/CFS and Long COVID, especially at tissue-specific sites.
- Persistent immune dysregulation in ME/CFS and Long COVID can account for the perpetuation of senescent endothelial cells by disenabling their clearance, and thus warrants the experimentation into the ability of patient leukocytes to eliminate senescent (endothelial) cells.
- Virus-induced endothelial senescence, potentially in genetically-susceptible individuals, has the potential to explain the symptoms and chronicity of ME/CFS and Long COVID, and hence warrants further investigation of vascular senescence in animal and human studies, as well as the advancement of cell-specific senescent biomarkers for clinical application.

## Introduction

Post-viral disease is an umbrella term used to describe a range of conditions that occur after viral infection, with long COVID and myalgic encephalomyelitis/chronic fatigue syndrome (ME/CFS) being the most studied. These diseases are characterized by a variety of symptoms that most commonly include unresolved fatigue, post-exertional malaise (PEM), cognitive dysfunction and brain fog, orthostatic intolerance, autonomic dysfunction, gastrointestinal disturbances, myalgia, neuralgia, sensory sensitivities, and others [1, 2]. Importantly, these symptoms persist long after acute infection.

The incidence of ME/CFS has increased over recent years [3] as a result of the COVID-19 pandemic, specifically due to the fact that a subset of patients who contract SARS-CoV-2 develop long-lasting symptoms, referred to as long COVID, that meet the diagnostic criteria for ME/CFS [4, 5], and as such, SARS-CoV-2 viral infection is a trigger for ME/CFS [6, 7]. Adding to the growing challenge of post-viral diseases is the fact that there are neither official mechanistic explanations for long COVID and ME/CFS, nor established diagnostic biomarkers and effective treatments. Elucidating the pathophysiological complexities associated with long COVID, ME/CFS, and other post-viral diseases is of paramount importance for clinical advancement in the field. From this, it is likely that effective diagnostic techniques and therapeutic strategies will result.

There are a number of proposed hypotheses to explain the induction and maintenance of ME/CFS and long COVID, some involving the central nervous system [8], the vasculature [9–12], viral persistence [13–15], immune dysregulation [16], autoantibody generation [17, 18], and the (neuro)endocrine system [19]. Anomalous clotting processes, characterized by the formation of heterogeneous (amyloid) deposits referred to as fibrinaloid complexes (microclots) and platelet dysregulation [20, 21], are features of both long COVID and ME/CFS [22–27]. It has, however, proven difficult to decipher cause and effect in this regard. In particular, the vascular component of long COVID and ME/CFS pathology has received much attention in recent years, especially due to the haematological and vascular consequences of SARS-CoV-2 infection, as well as the virus’s predilection for endothelial cells [28]. There is extensive evidence pointing to endothelial dysfunction in both long COVID [29–37] and ME/CFS [12, 29, 38–44]. Consequentially, many hypotheses regarding the pathogenesis of ME/CFS and long COVID are centred around endothelial cell dysfunction, often along with downstream consequences of other systems [9, 10, 17, 32, 45–47]. Blood flow dynamics are perturbed in both long COVID and ME/CFS cohorts, with major findings revolving around reduced cerebral blood flow [48–50] and reduced perfusion of certain brain regions and organs [51–55].

Here, we elaborate on the endothelium being a primary site of disease induction and maintenance through a process of endothelial senescence. With this, we discuss a novel hypothesis that can explain the multisystem and chronic nature of ME/CFS and long COVID. We review the evidence of endothelial senescence as the functional culprit of ME/CFS and long COVID. Furthermore, we provide a link between acute infection and the induction of ME/CFS and long COVID, and between vasculature-related disease mechanisms and immune dysfunction that are typically observed in long COVID and ME/CFS.

### Senescence and associated mechanisms

Cellular senescence is one of the hallmarks of aging, and describes a state of stable cell cycle arrest combined with other molecular, morphological, and metabolic changes. The response of cells to enter a senescence programme occurs following persistent and unresolved stress leading to cellular damage and dysregulation [56]. This can be due to replicative exhaustion, called replicative senescence, in which continuous shortening of the telomeres towards a critically short length signals for chronic activation of the DNA damage response and activation of cell cycle arrest pathways [57]. Other senescence triggers, such as hyperactivation of oncogenes (e.g., Ras), drive oncogene-induced senescence [58]; such programmes have likely evolved as a cell-intrinsic mechanism to suppress cell proliferation and tumour formation [59]. Radiation, radiomimetic drugs, genotoxins, or reactive oxygen species (ROS) can all lead to excessive DNA damage, driving DNA damage-induced senescence [60]. Induction of senescence through viral infection is a more recent phenomenon which clearly plays a role in the pathology of several viral diseases.

Although there is no single unique biomarker of senescence, senescent cells can be detected by their presentation of a panel of senescence-associated features. Exit from the cell cycle is identified by the high expression of cyclin kinase inhibitors, *CDKN1A* and *CDKN2A*, encoding p21 and p16, respectively, which establish and maintain cell cycle arrest [61]. Senescent cells exhibit profound nuclear alterations including persistent DNA damage which are distinct from acute DNA lesions, termed DNA-SCARS (DNA Segments with Chromatin Alterations Reinforcing Senescence [62]), chromatin changes (termed senescence-associated heterochromatin foci, SAHFs [63]) and loss of nuclear membrane integrity underpinned by decreased lamin B1 expression, leading to cytoplasmic chromatin fragments (CCFs) [64]. Senescent cells exhibit strong upregulation of anti-apoptotic pathways, which explains their resistance towards programmed cell death despite their high levels of internal cellular damage.

Organelle dysfunction is also highly apparent in senescent cells, and includes disrupted mitochondrial ATP production and lysosomal hypertrophy [65]. Mitochondrial dysfunction of senescent cells has been demonstrated to be essential for their hypersecretory phenotype, known collectively as the senescence-associated secretory phenotype (SASP) [66]. The high lysosomal load of senescent cells leads to high levels of the lysosomal enzyme, β-galactosidase, even at suboptimal pH 6.0 [67].

The SASP encapsulates a pro-inflammatory and tissue remodelling secretome [66], though its exact components depends on the cell type, mode of senescence induction (e.g., replicative, DNA damage, oncogene-induced), and stage of senescence (e.g., early, middle, or late) [68]. This phenotype is highly immunogenic (containing, for example, interleukin (IL)-6, IL-8, and tumour necrosis factor (TNF)-α)); thus, senescent cells attract immune cells, such as CD8+T cells, natural killer cells, and neutrophils, to coordinate their removal [69, 70]. Hence, senescence is a physiological state that evolved to aid tumour cell removal and tissue remodelling [59]. Additionally, the SASP drives paracrine senescence of local cells, which may provide another tumour-suppressive mechanism that restricts cancerous transformation [71].

Senescence and virus infection are bidirectional and hence self-reinforcing processes, whereby viral infection can drive senescence, and senescence predisposes to further viral infection. Vascular endothelial senescence is apparent in lung autopsy samples from patients with severe COVID-19 [72] and is likely involved in the pathophysiology of COVID-19. For example, replicative senescent endothelial cells are especially susceptible to SARS-CoV-2 infection compared to non-senescent cells [73]. Importantly, SARS-CoV-2 viral proteins degrade host cell components of the DNA damage response, ablating the cell’s DNA repair capacity and driving cell senescence [74]. Cultured media from virus-induced senescent fibroblasts can induce senescence in human endothelial cells, highlighting an important role of bystander or paracrine senescence after virus infection through the SASP [75]. Viruses therefore likely evolved to exploit the senescence programme to exacerbate their propagation across tissues. For example, the SASP of irradiation-induced senescent endothelial cells increases expression of viral entry proteins, ACE2 and TMPRSS2, in SARS-CoV-2-permissive human lung epithelial cells, an effect which can be blocked with a neutralising anti-IL1α antibody [76]. Moreover, treatment with senolytic drugs that remove senescent cells by targeting their anti-apoptotic pathways improved mouse survival from coronavirus infections, thus underscoring the importance of senescent cells in driving acute disease pathology [75–77].

### Endothelial senescence as an explanation for the induction and maintenance of ME/CFS and long COVID

Recently, we discussed the role of a dysregulated endothelium and vasculature in explaining ME/CFS symptoms, and how herpesvirus infection can drive vascular dysfunction [46]. Latent endothelial cell infection might be sufficient to induce symptoms associated with ME/CFS, and particularly, its organ-specificity is an important factor for symptom manifestation.

**Fig. 1:**
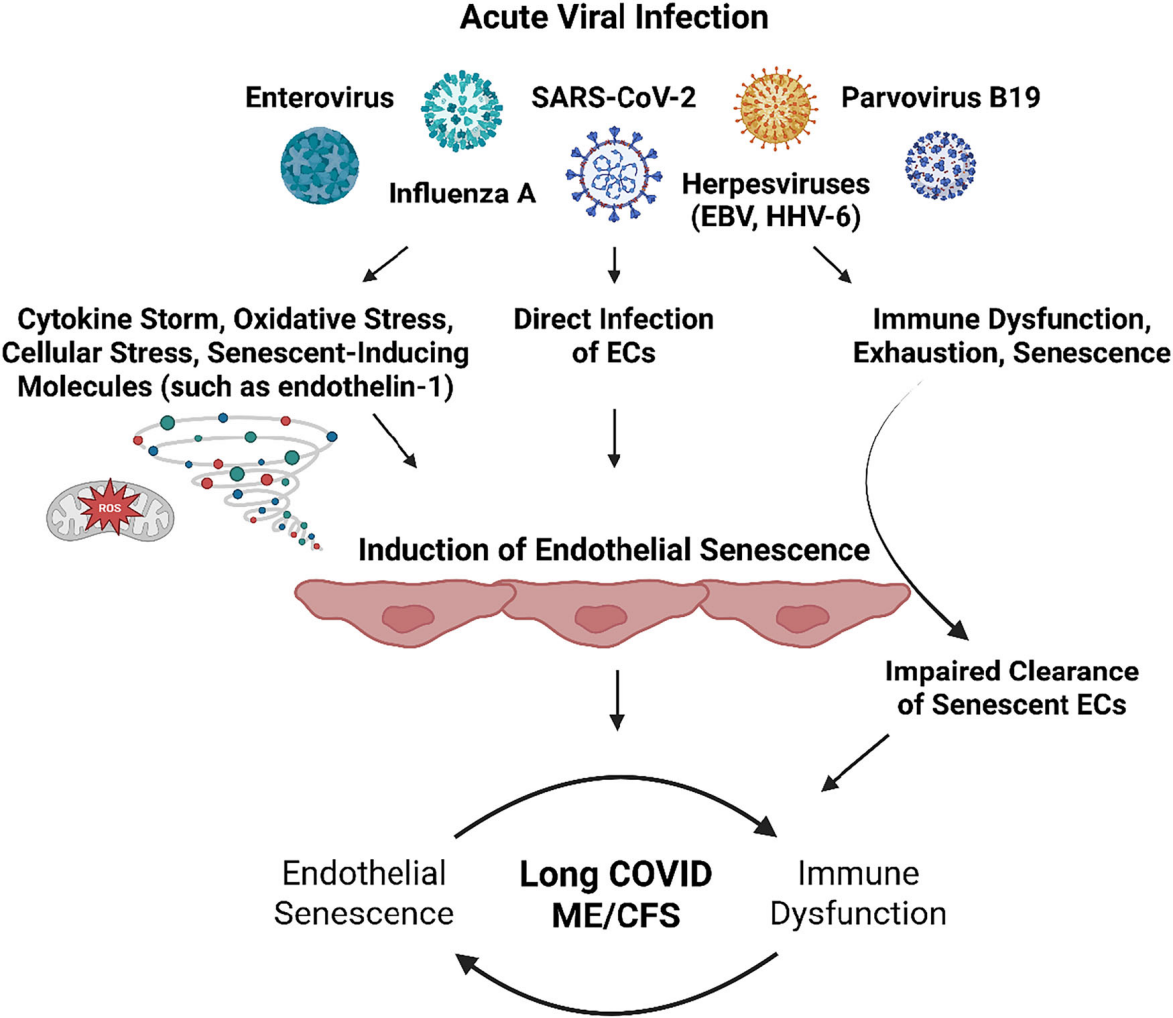
Overview of the present hypothesis. Acute viral infections induce endothelial senescence via direct and indirect mechanisms, which is then sustained by immune dysfunction and impaired clearance of senescent endothelial cells. (EC - endothelial cell). (Created with the paid version of Biorender.com).

While there is currently no causative proof of this hypothesis, here we present an expanded role of viral infections in promoting chronic endothelial dysfunction and senescence. We propose that viral infection results in direct and indirect endothelial modulation resulting in long-term endothelial senescence. These senescent endothelial cells can persist for months to years after the acute infection, sustained by a dysfunctional immune system (discussed later; Fig. 1).

The burden and organ-location of endothelial senescence seems crucial; for example, senescent endothelial cells within specific arteries (e.g., carotid and vertebral arteries) can explain reduced cerebral blood flow (and other perfusion deficits) observed in patients due to their impaired regulation of the vasculature in response to perfusion requirements, which can subsequently cause neurological symptoms as a result of impaired oxygenation, glucose supply, and waste clearance; senescent endothelial cells of the blood-brain barrier (BBB) can also inform the neurological symptoms associated with ME/CFS and long COVID; senescent endothelial cells that reside by skeletal muscle and the consequences of impaired perfusion might be able to explain bioenergetic disturbances in myocytes, observed myopathies, exercise-intolerance, and PEM; a senescent endothelium in the gastrointestinal tract will lead to impaired barrier function, gut dysbiosis, and potentially microbial translocation into circulation and other tissues [45]. Indeed, senescent endothelial cells and other vascular cells, in particular, have the potential to cause systemic physiological dysfunction. This pathology has the potential to explain the persistence of pathological mechanisms (Fig. 1), such as vascular dysregulation and perturbed blood flow, immune recruitment and dysregulation, persistent clotting pathology, platelet dysregulation, increased intestinal permeability, and impaired BBB function, as well as the persistence of symptoms and the multisystem nature of ME/CFS and long COVID.

It is necessary to ascertain whether the viruses implicated in ME/CFS and long COVID, including SARS-CoV-2, EBV, HHV-6, influenza A virus, enteroviruses, parvovirus B19, and potentially others, are capable of infecting endothelial cells and causing endothelial dysfunction. Indeed, these viruses do have the ability to infect endothelial cells (Table 1). Importantly, SARS-CoV-2 and influenza A virus can induce endothelial senescence [72, 75, 78–80]. These results indicate that the aforementioned viruses can indeed infect endothelial cells, although more studies are required to assess the potential of these viruses, in addition to SARS-CoV-2 and influenza A, in inducing endothelial senescence, specifically.

**Table 1:** Evidence of viruses implicated in ME/CFS (and long COVID) in causing direct infection and induction of senescence specifically in endothelial cells.

Virus type	Direct infection of endothelial cells	Induction of endothelial senescence	References
**SARS-CoV-2**	Co-localisation with endothelial cells in autopsied human and mouse tissue; Infection of human lung and brain endothelial cells.	Upregulation of p21 in endothelial cells from autopsied human lung tissue; Infected macaques exhibit increased p16 in lung endothelial cells; Upregulation of p21 when exposed to infected epithelial cells and culture media, and pseudovirus expressing spike protein; Spike-induced upregulation of p16 and p21.	[72, 78–80, 224–232]
**EBV**	EBV identified in human endothelial cells in situ; Infection of human brain, dermal, and umbilical vein endothelial cells; Endothelial cells uptake EBV-infected lymphocytes.	Unknown	[149, 233–237]
**HHV-6**	Infection of human umbilical vein, aortic, lymphatic, and microvascular endothelial cells; HHV-6B genome discovered integrated into an endothelial cell line derived from umbilical cord tissue.	Unknown	[238–243]
**Influenza-A**	Infection of human lung, brain, and umbilical vein endothelial cells.	Upregulation of p16 and p21 in endothelial cells in vitro; Endothelial cells of infected mice and macaques exhibit p16 expression.	[154, 244–251]
**Enteroviruses**	Infection of human microvascular, umbilical vein, cardiac, and brain endothelial cells.	Unknown	[152, 153, 252–254]
**Parvovirus B19**	Infection of placental endothelial cells from human tissue; Infection of various tissue-specific endothelial cell lines.	Unknown	[255, 256]

While we are focusing solely on endothelial cells, it deserves mentioning that there is evidence, albeit not cited here, of senescence induction in non-endothelial cells for these viruses.

With regards to SARS-CoV-2, increased levels of chitotriosidase and stathmin 1 were found in plasma samples from hospitalised COVID-19 patients 3 months after infection [81], which are suggestive of the induction of cellular senescence. Indications of T-cell senescence have also been observed in long COVID patients [82] and COVID-19 survivors [83]. There is a clinical trial recruiting patients for the investigation of senescent cells (cell type not specified) and their secretome in long COVID patients (NCT ID: NCT04903132).

Direct viral infection of endothelial cells is not a prerequisite for senescence induction, as inflammatory cues and oxidative stress, as well as some specific molecules, such as endothelin-1 (ET-1), SARS-CoV-2 spike protein, leukocyte microparticles, angiotensin II, homocysteine, cytokines, and others [79, 84–87], have the potential to induce endothelial senescence. Hence, induction of post-viral diseases might be independent of endothelial cell viral infection.

Sfera et al. [45] published hypotheses involving gastrointestinal-associated endothelial senescence as an important pathological component of ME/CFS and long COVID pathology [45, 88]. These studies focussed on the intestinal barrier, where endothelial senescence enables the subsequent translocation of microorganisms and inflammagens (such as LPS) into circulation and tissues, including skeletal muscle and central nervous system, which can then give rise to pathology. Although the present hypothesis encompasses the systemic endothelium, as well as tissue-specific sites such as arteries, neurovasculature, and skeletal muscle, and not only the gastrointestinal tract, Sfera et al. offered mechanistic reasoning for the induction of endothelial senescence via SAR-CoV-2-induced upregulation of angiotensin II [88].

### Endothelial senescence-associated secretory phenotype (SASP) in ME/CFS and Long COVID

**Fig. 2:**
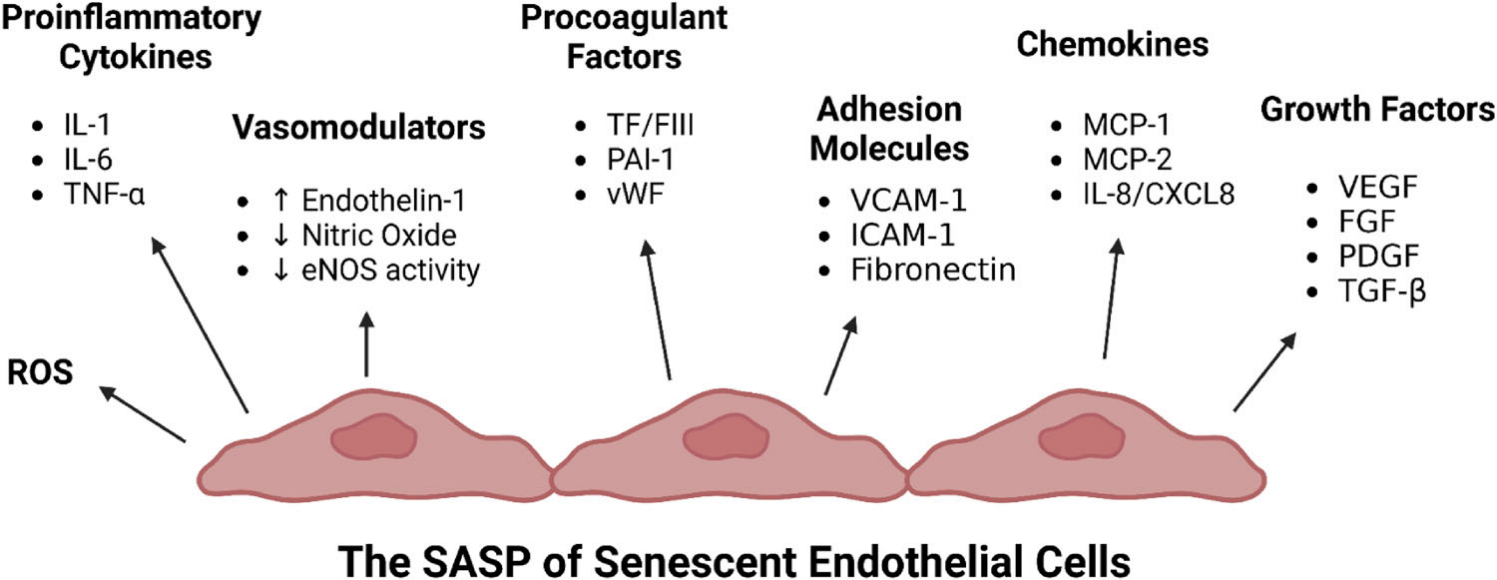
The senescence-associated secretory phenotype (SASP) of senescent endothelial cells. Senescent endothelial cells and their associated SASP is one that is proinflammatory, procoagulant, and prooxidative, and upregulates the expression of adhesion molecules, chemokines, and growth factors. Furthermore, and important to the findings of perturbed blood flow and perfusion deficits in long COVID and ME/CFS, the expression of vasomodulators is dysregulated in senescent endothelial cells, which predominantly manifests as a vasoconstriction effects and subsequent blood flow and perfusion deficits (uncoupling between ET-1 and NO). (Created with the paid version of Biorender.com).

The consequences of endothelial senescence [89] in a micro- and macrovascular context have been reviewed [90], with a specific focus on their proinflammatory, pro-oxidant, procoagulant, and vasoconstrictor effects [91]. Endothelial senescence is characterized by a specific profile of expressed and secreted molecules, the endothelial SASP (Fig. 2). This is characterized by: an increase in proinflammatory cytokines including IL-1β, IL-6, and tumour necrosis factor-α (TNF-α); an increase in procoagulant factors including tissue factor (TF) [92], plasminogen-activator inhibitor-1 (PAI-1) [93, 94], and von Willebrand factor (vWF) [95]; a dysregulation of vasomodulators including reduced nitric oxide (NO) and increased endothelins [96]; an increase in adhesion molecules including intercellular adhesion molecule-1 (ICAM-1), vascular cell adhesion molecule-1 (VCAM-1), and fibronectin [94]; an increase in chemokines including monocyte-chemoattractant protein-1 (MCP-1)/C-C motif ligand 2 (CCL2), MCP-2, IL-8/chemokine (C-X-C motif) ligand 8 (CXCL8); an increase in growth factors including vascular endothelial growth factor (VEGF), fibroblast growth factor (FGF), platelet-derived growth factor (PDGF), transforming growth factor-β; and an increase in ROS production [89, 97–99].

Specific endothelial markers that can be indicative of their senescence have been shown to be increased in ME/CFS and long COVID cohorts, including ET-1 [29, 31, 43, 44, 100, 101], VEGF-A [102], VCAM-1 [44, 100], ICAM-1 [31, 103], PAI-1 [44], IL-8 [31], MCP-1 [31] and FGF-2 [102]. Elevated levels of ET-1 in long COVID, but also during acute COVID-19 [104–106], are known to induce endothelial senescence [84, 89]. A variety of other molecules or processes (e.g., inflammation, oxidative stress, signalling molecules, intracellular cues, etc.) can also induce endothelial senescence independent of ET-1 [99, 107]. In conclusion, there is experimental evidence suggestive of endothelial senescence in long COVID and ME/CFS, and that many of these upregulated molecules, such as ET-1, are in turn capable of inducing endothelial senescence. Further investigation into the burden of senescence endothelial cells and the SASP in long COVID and ME/CFS is thus warranted.

### Senescent endothelial cells and vascular dysfunction

Hypotheses and inferences regarding perfusion deficits and vascular abnormalities in ME/CFS and long COVID are based upon the evidence of predominant manifestation of vasoconstriction, enhanced sympathetic activity, and perturbed blood flow and perfusion [9, 10, 48–51, 108–110]. We suggest that endothelial senescence (and potentially the dysfunction and senescence of other vascular cells, including smooth muscle cells) contributes to these mechanisms and symptoms.

The SASP associated with senescent endothelial cells is a profile, in the context of vascular regulation, that favours the effects of vasoconstriction [89, 90, 111]. Indeed, perfusion deficits and vascular abnormalities in ME/CFS and long COVID are often related to vasoconstriction and enhanced sympathetic activity [9, 10, 48–51, 108–110]. An increase in ET-1 and a concurrent decrease in NO, particularly due to a reduced eNOS activity, is associated with the SASP [96]. These phenomena have consequences for the regulation of blood flow and hence provides a mechanistic link between endothelial senescence and the reduced cerebral blood flow underlying orthostatic intolerance in long COVID [50, 109] and ME/CFS [48, 49, 54] (Fig. 1).

Senescent endothelial cells exhibit phenotypic changes and increased adhesive properties to the basement membrane, which impact haemodynamics [112], and also display increased permeability caused by compromised barrier integrity [113, 114]. The endothelial SASP is associated with age-related arterial dysfunction and stiffness [90, 115], and hence can explain blood flow perturbations and perfusion deficits observed in blood vessels of patients, e.g., the carotid and vertebral arteries. There are also signs of cardiac abnormalities and inefficiencies in ME/CFS and long COVID [116, 117], which can be explained, in part, by endothelial senescence [89].

Since endothelial senescence affects both systemic and microcirculation [89, 90], it raises the question of whether impaired blood flow regulation and subsequent perfusion deficits can contribute to the multisystem involvement observed in ME/CFS and long COVID, as well as the manifestation of non-specific symptoms. Vascular abnormalities, including blood flow disturbances and perfusion deficits, may help explain symptoms across various physiological systems, accounting for both specific and non-specific symptoms [9, 10]. For instance, this systemic dysregulation of blood flow, along with other pathological effects of endothelial senescence, could contribute to the persistent, unresolved fatigue (and exercise intolerance) experienced by patients. Relevantly, there is an association between endothelial dysfunction and (cognitive) fatigue [118].

### Proinflammatory component of the endothelial SASP and immune dysfunction, exhaustion, & senescence—A possible explanation for the maintenance of endothelial senescence in ME/CFS and long COVID

The SASP of senescent endothelial cells recruits and activates immune cells via the expression of cytokines, chemokines, and adhesion molecules [119]. These processes, along with impaired barrier function and increased permeability [114], may lead to immune dysregulation and tissue infiltration of immune cells. Senescent endothelial cells promote immune exhaustion and senescence [120], which is also implicated in patients with ME/CFS and long COVID [20, 121]. However, senescence of leukocytes likely also occurs independently of senescent endothelial cells during SARS-CoV-2 infection [122].

In acute COVID-19, there are various studies reporting indications of immune exhaustion and immunosenescence in patients [123, 124], as well as in long COVID [82, 125]. While it is unknown whether immunosenescence occurs in patients with ME/CFS [126], there are indications of immune exhaustion [20, 126]. The idea that senescent immune cells alone can give rise to the myriad symptoms associated with ME/CFS and long COVID is unconvincing, but that is not to say that immune deficits are negligible in ME/CFS and long COVID pathology—it is plausible that such immune deficits sustain the primary pathology of ME/CFS and long COVID. In the context of the present hypothesis, these immune deficits might help to sustain endothelial senescence.

Natural killer (NK) cells are involved in clearing senescent cells [127, 128], but their cytotoxicity is reduced in patients with ME/CFS [129, 130]. Neutrophil extracellular traps (NETs) implicated in long COVID [131, 132], which are released from activated neutrophils in response to senescent endothelial cells, can aid in endothelial remodelling [133] and are also structurally associated with heterogeneous fibrinaloid microclots [134]. Along with NK cells and neutrophils, macrophages and T-cells can also eliminate senescent cells [128]. Macrophages and CD8+T-cells also exhibit functional deficits in ME/CFS and long COVID and correlate with symptoms [20, 135, 136]. Furthermore, the complement system aids in removal of senescent cells [137], and complement dysfunction is implicated in the pathophysiology of ME/CFS [12, 138] and long COVID [139]. Lastly, dysfunctional immune cells are also capable of inducing endothelial senescence [140], and endothelial cells can function to clear defective and senescent leukocytes [141]. The evidence of immune exhaustion in ME/CFS and long COVID and immune senescence in long COVID, together with endothelial senescence, can explain the myriad symptoms and chronicity of ME/CFS and long COVID.

**Fig. 3:**
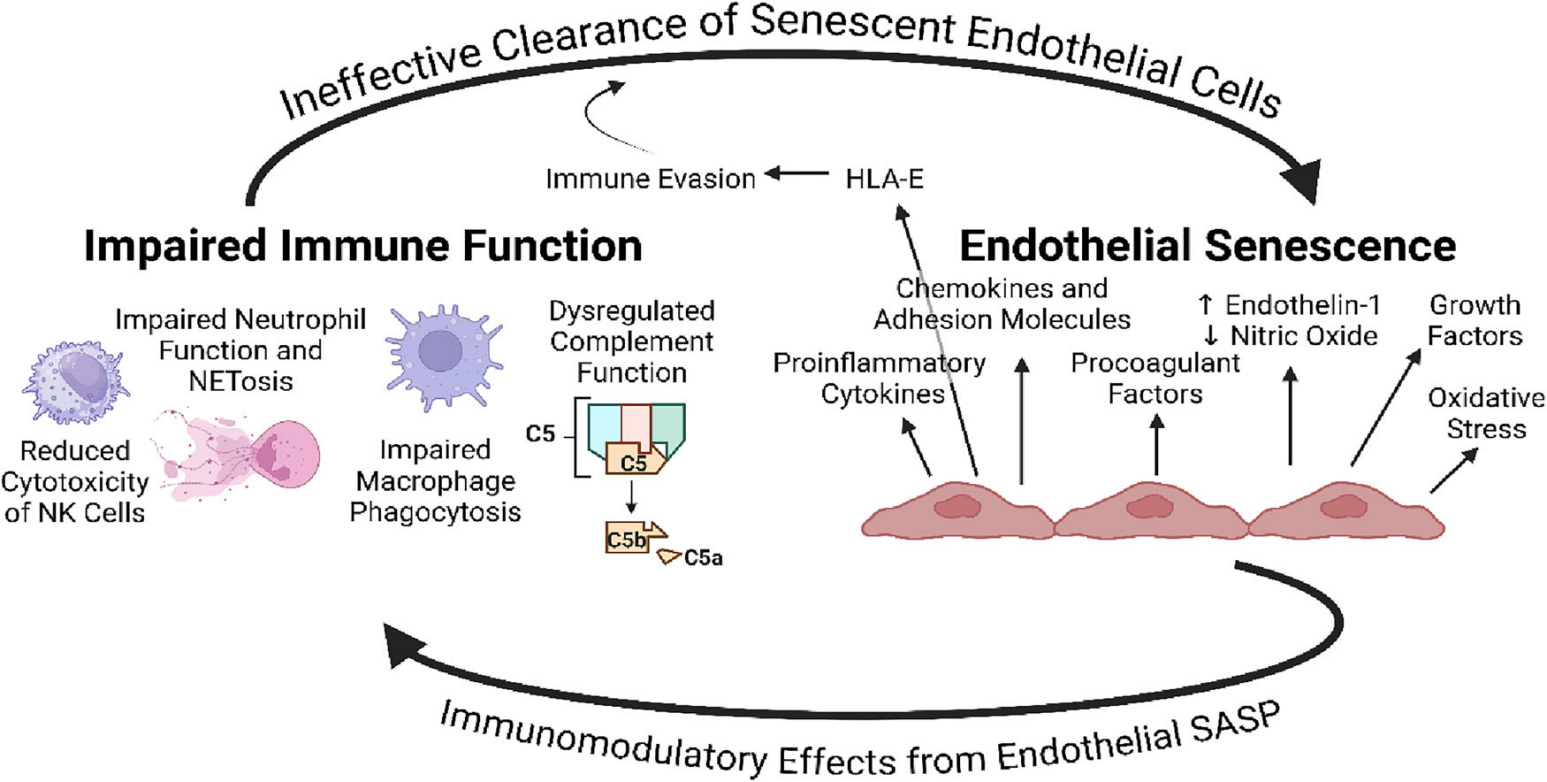
Maintenance of endothelial senescence by impaired immune function and expression of HLA-E. Immune dysfunction, including reduced NK cell cytotoxicity, impaired macrophage phagocytosis, dysregulated NETosis, and shortcomings in complement function—which, incidentally, are common features of ME/CFS and Long COVID—lead to ineffective clearance of senescent cells, enabling their persistence and expression of the SASP over long periods of time. In turn, the endothelial SASP exerts immunomodulatory effects that induce immune activation and recruitment, tissue infiltration, and leukocyte dysfunction and exhaustion, along with vasoconstriction effects, immunothrombosis, and impaired tissue healing and repair. Furthermore, senescent endothelial cells express HLA-E, which evades NK and CD8+T-cell responses. (Created with the paid version of Biorender.com).

Considering the role of natural killer cells, neutrophils, T-cells, macrophages, and the complement system in clearing senescent endothelial cells, the impaired function of these immune cells and systems in ME/CFS [136] and long COVID [142] offer an explanation for the maintenance of endothelial senescence, linking it bidirectionally with immune dysfunction, i.e., endothelial senescence induces immune dysfunction, and dysfunctional immune cells enable the sustainment of endothelial senescence long past the acute infection. Additionally, senescent fibroblasts evade immune responses by expressing the major histocompatibility complex molecule HLA-E, which normally inhibits NK cell and CD8+T-cell responses via engagement of the immune inhibitory natural killer group 2A (NKG2A) receptor, thereby preventing leukocyte cytotoxicity and hindering the removal of senescent cells [70]. Indeed, it has been shown that irradiation-induced senescent endothelial cells also express HLA-E, which enables their evasion of NK cell cytotoxicity [143]. Hence, this provides another mechanism by which senescent endothelial cells may persist and can direct future experiments investigating this hypothesis (Fig. 3).

### Senescence of brain microvascular endothelial cells and neurological symptoms

Brain microvascular endothelial cells (BMECs) form the essential and highly selective BBB, which plays a crucial role in maintaining homeostasis of the central nervous system. However, senescent BMECs may contribute to age-related dysfunction and the breakdown of the BBB [144], potentially leading to neurovascular issues and thus cognitive impairment, as well as an increased risk of neurological disorders. Senescent BMECs show signs of loss of barrier integrity and increased permeability [113], as well as perturbed mitochondrial function and overall cellular bioenergetics [145]. They also impair the regulation of microglia and can result in dysregulated inflammatory sequelae in the central nervous system [146].

Using SARS-CoV-2 as an example again, this virus has the ability to directly infect BMECs [147, 148] and hence supports the current hypothesis and notion of BMEC senescence as a result of acute viral infection. Additionally, there is evidence of herpesvirus infection of BMECs [149] and infection of the central nervous system in ME/CFS [150], as well as evidence of BMEC infection by enterovirus [151–153] and influenza A virus [154, 155]. Senescence of BMECs and the local and systemic ramifications that ensue can explain cognitive impairment and other neurological issues associated with ME/CFS and long COVID—indeed, many recent studies have shown that endothelial senescence can bring about cognitive dysfunction [111, 132]. To add, peripheral endothelial senescence has been shown to have an impact on BBB integrity and cognition [156] and hence, neurological symptoms might also occur due to peripheral endothelial senescence. Interestingly, ET-1—released by senescent endothelial cells and has been found to be upregulated in ME/CFS and long COVID (as discussed earlier)—is associated with cognitive dysfunction [157]. Hence, the effects of endothelial senescence impact not just blood flow regulation and tissue perfusion in the periphery, but also the central nervous system.

Importantly, disruption of BBB integrity and function has been demonstrated in long COVID cohorts [158, 159] and have been included in several hypotheses for ME/CFS [8, 108, 160]. The proinflammatory profile of BMECs SASP is one that supports various neuro-centred hypotheses of ME/CFS and long COVID [8, 53, 108], which encompass neuroinflammation, BBB disruption, and impaired perfusion, and seek to link such mechanisms to (neurological) symptoms. Hence, the neurovascular and neuroinflammatory mechanisms of ME/CFS and long COVID can be a result of endothelial senescence, thereby giving rise to cognitive deficits and other neurological symptoms.

The findings of herpesvirus RNA and protein in the central nervous system of ME/CFS patients [150] can be a reflection of both impaired immune function and impaired BBB integrity, with the latter brought about by senescence of BMECs. Mechanism-focused reviews on BMEC senescence and the consequences thereof for both neurological and peripheral function are published [111, 132, 144, 156]. Of course, in light of reduced cerebral blood flow, senescence of BMECs might be secondary in the contribution to neurological symptoms in ME/CFS and long COVID. Perfusion deficits in various brain regions in ME/CFS and long COVID [52, 54, 161] might be a result of a local burden of senescent BMECs.

### Endothelial senescence and gastrointestinal symptoms

ME/CFS and long COVID cohorts demonstrate a variety of gastrointestinal disturbances and symptoms [162, 163]. There is evidence of gut dysbiosis in ME/CFS [164] and long COVID [165], coupled to increased intestinal permeability and microbial translocation in both diseases [166, 167]. Relevantly, several hypotheses of disease induction and maintenance are centred around gut integrity and microbiota [168, 169].

Sfera et al. have published on the role of endothelial senescence in a gastrointestinal context, and the subsequent ramifications for intestinal barrier integrity and of microbial (and LPS) translocation into circulation [45]; hence, the gastrointestinal aspect of this theory is not elaborated further here. Ultimately, endothelial senescence at the intestinal border can lead to loss of barrier integrity and increased gut permeability, subsequently enabling microbial translocation, which can promote inflammation and tissue damage in the systemic vasculature and organs (Fig. 1).

To add, there is evidence that gut dysbiosis and some microbial metabolites, such as trimethylamine-N-oxide and phenylacetic acid, can induce endothelial senescence and contribute to systemic vascular pathology in this context [170, 171]; hence, the bidirectional consequences of endothelial senescence and gut dysbiosis are of interest in the context of the present hypothesis.

### Senescence of endothelial cells and post-exertional malaise

One of the more debilitating symptoms of patients with ME/CFS and long COVID is PEM, which is the worsening of symptoms or development of new symptoms in response to physical or psychological exertion. Unlike fatigue, this is typically a delayed reaction, but one that can last for hours or days. We argue here that the impact of physical exertion on a dysfunctional, maladaptive, and a specifically senescent endothelium is enough to induce a significant systemic response that gives rise to PEM.

The transition from rest to exercise demands a rapid and tightly coordinated increase in oxygen delivery in order to accommodate the increased metabolic demand of the working skeletal muscle tissue [172]. These increases in oxygen and nutrient delivery are accomplished by sympathetic signalling, increasing the cardiac output, breathing frequency, but also microvascular regulation, all promoting additional oxygen extraction from the blood [173]. Invasive cardio-pulmonary exercise testing in both ME/CFS and long COVID patients, however, has indicated a peripheral problem of impaired oxygen extraction during exercise, as cardiac and pulmonary function were less affected [174, 175]. Malfunctioning endothelial cells may predispose to perturbations in efficient oxygen and nutrient exchange, especially upon exertion, affecting oxygen perfusion and diffusion and local tissue oxygenation. Also, by-products build up in skeletal muscle tissues causing cell acidification occurs following exertion. The insufficiency of a senescent endothelium to efficiently dispose of these exertion-induced waste products worsens exertion and local muscle pain. This skeletal muscle pathology is likely further exacerbated by the SASP-induced dysregulation of immune cells and inflammatory responses.

These dynamic perfusive responses are governed partly by the endothelium, which rapidly adjusts vascular tone through both mechanical and chemical cues. However, endothelial senescence may severely impair vasodilation, as the SASP is marked by a predominance of vasoconstrictors over vasodilators. The SASP is associated with an increased expression of ET-1, and reduced eNOS and NO expression. ET-1 is indeed significantly higher in ME/CFS [29, 43, 44, 100] and long COVID [29, 31, 44, 100, 101] patients, along with some initial evidence of decreased NO expression and eNOS function in ME/CFS [41, 172, 176]. This vasomotor imbalance is especially harmful during exercise, as increased blood flow and the resulting shear stress should induce vasodilation to ensure proper tissue perfusion. This is further supported by the important role of ET-1 in regulating blood flow dynamics during exercise [177–179], as it is associated with lower oxygen consumption during exercise [180].

ET-1 also exerts direct effects on skeletal muscle cells and tissue. Beyond its previously addressed role as vasoconstrictor, ET-1 can also bind to the endothelin B receptor on myoblasts and hinder skeletal muscle myogenesis [181]. The same study also noted that ET-1 reduces the expression of MyoD, MyoG, and MyHC on cultured myoblasts and of MyHC in differentiated myotubes. Proinflammatory sequelae, including the expression of IL-6 and TNF-α, also ensue in myoblasts exposed to ET-1 [182]. Glucose uptake is impaired in skeletal muscle cells exposed to ET-1, via the inhibition of Akt [183]. In addition to ET-1 causing endothelial senescence, ET-1 can induce fibrosis (exemplified via the expression of fibronectin) in cultured myoblasts via ROS production and activation of the PI3K-AKT-GSK pathway [184]. Indeed, increased levels of fibronectin in ME/CFS and long COVID have been reported [185] as well as thickening of the capillary basement membranes in long COVID [186, 187], with the latter emphasizing possible substance exchange issues. Together, these can help with explaining the pathophysiology of muscle-specific alterations in ME/CFS and long COVID.

PAI-1, another component of the endothelial SASP, can exert deleterious effects on skeletal muscle cells and tissue [188]. Most notably, it inhibits skeletal myogenesis and promotes fibrosis [188], ultimately compromising skeletal muscle regenerative capacity. Indeed, senescent endothelial cells are associated with myopathy in dystrophic skeletal muscle in mice [189]. Moreover, senescent markers (although not identified to be from endothelial cells) are upregulated in skeletal muscle tissue from patients with Duchenne muscular dystrophy [190].

Senescent endothelial cells exhibit an impaired healing ability, which implicates vascular and surrounding tissues [89, 191]. Although some components of the SASP, such as PDGF-AA, have been shown to be beneficial in tissue repair [97], this is in the context of injury and the subsequent acute response, and not from the perspective of virus-induced senescence and the persistence of senescent endothelial cells. Endothelial cells form part of the microenvironment from which satellite cells and myocytes receive constant signalling and hence regulation, and secrete growth factors, angiopoietin-1, and microRNAs that can activate or inhibit satellite cells [192, 193]. How these cells communicate with each other, and how exercise worsens this inter-cellular communication, is currently unknown.

**Fig. 4:**
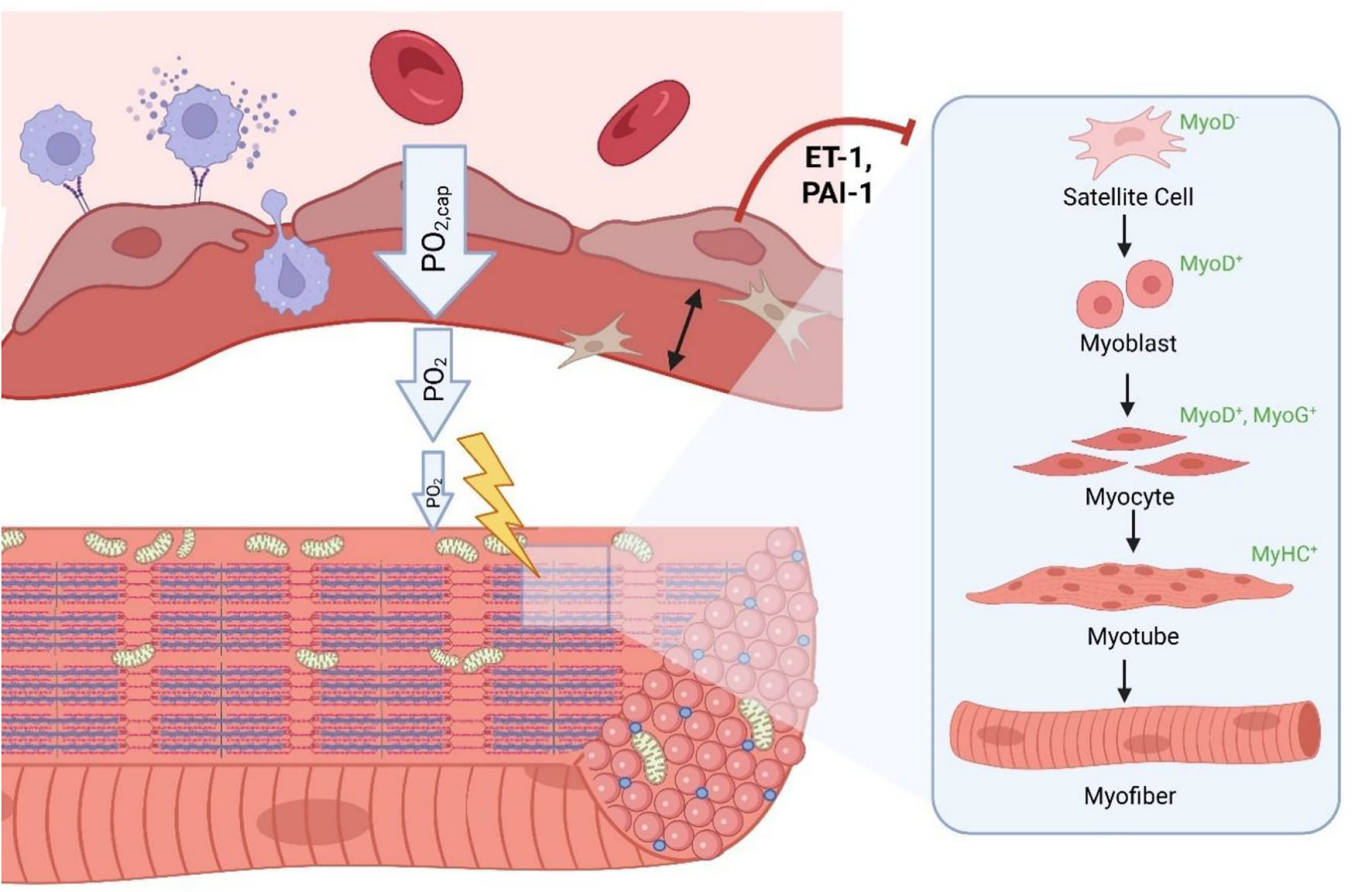
Effects of endothelial senescence on skeletal muscle tissue. Uncoupling between perfusion requirements and blood flow, as well as basement membrane thickening impeding the diffusion barrier, are primary to molecular and cellular dysregulation at tissues. In theory, the resulting oxygenation deficit could lead to local hypoxia, as well as the accumulation of waste products upon exercise. Combined with immune impairments, this may result in further disruptions of the structural and functional integrity of skeletal muscle tissue. The senescent immune environment may additionally inhibit skeletal muscle regeneration upon injury, via elevated levels of ET-1 and PAI-1, impairing various steps of myogenesis. (Created with the paid version of Biorender.com).

In conclusion, a senescent endothelium will impair nutrient delivery to and waste removal from skeletal muscle tissue. Senescent endothelial cells and their consequences on local blood flow, perfusion, and immune function are not conducive to effective tissue supply and waste removal, and tissue remodelling and repair [11]; hence these factors can lead to pathological insult upon exertion and impaired muscle regeneration (Fig. 4). These effects can result in a delayed recovery response upon exercise and an exaggerated intolerance to a second exercise insult if conducted in close succession to the first, i.e., the second exercise intervention will result in impairments greater than those observed in the first exercise intervention—this is reflected in consecutive exercise regimens involving cardiopulmonary exercise tests in ME/CFS [194, 195].

### Procoagulant factors of senescent endothelial cells and prolonged coagulation abnormalities

Abnormal clotting, including heterogeneous fibrinaloid microclots and dysregulated platelets, are major features of long COVID and ME/CFS. Heterogeneous fibrinaloid microclots can be induced by minuscule amounts of suitable trigger molecules [196–199]. We have previously noted increased levels of heterogeneous microclots, dysregulated platelets, abnormal clotting kinetics [22], along with dysregulated coagulation and endothelial markers [12]. Other groups have also found dysregulated platelets in ME/CFS cohorts [25, 200, 201]. Furthermore, with the evidence of a dysfunctional endothelium in ME/CFS and long COVID, an impairment in the regulation of coagulation is expected.

Senescent endothelial cells and their proinflammatory SASP prompt thrombotic processes [202] by cytokine- and chemokine-mediated recruitment of platelets which can lead to dysregulated clot formation and platelet levels [203–205], and by releasing procoagulant factors such as TF [92, 205] and vWF [95]. Silva et al. [205] inferred that the release of TF was dependent on NAPH-oxidase- and cyclooxygenase 2-induced oxidative stress, and also demonstrated that such procoagulant activity in senescent endothelial cells was blunted with NAPDH oxidase and cyclooxygenase 2 inhibitors [205]. Furthermore, PAI-1—expressed as part of the endothelial SASP [93]—can help to explain the persistence of heterogeneous fibrinaloid microclots and other clotting abnormalities in patients, as elevations in PAI-1 leads to a reduction in activated plasmin and therefore a reduction in fibrinolysis.

It has also been shown that atherosclerotic plaque-derived microparticles induce endothelial senescence in cultured endothelial cells and promote the expression of tissue factor and procoagulant microparticles, as well as a reduced expression of NO via the downregulation of eNOS [206]. In ME/CFS, plasma from ME/CFS patients downregulates NO expression in healthy endothelial cells [176], highlighting the role of plasma-derived substances and extracellular vesicles in altering vascular function and offering a target for therapy. The link between endothelial senescence and thrombosis has been discussed [207].

**Fig. 5:**
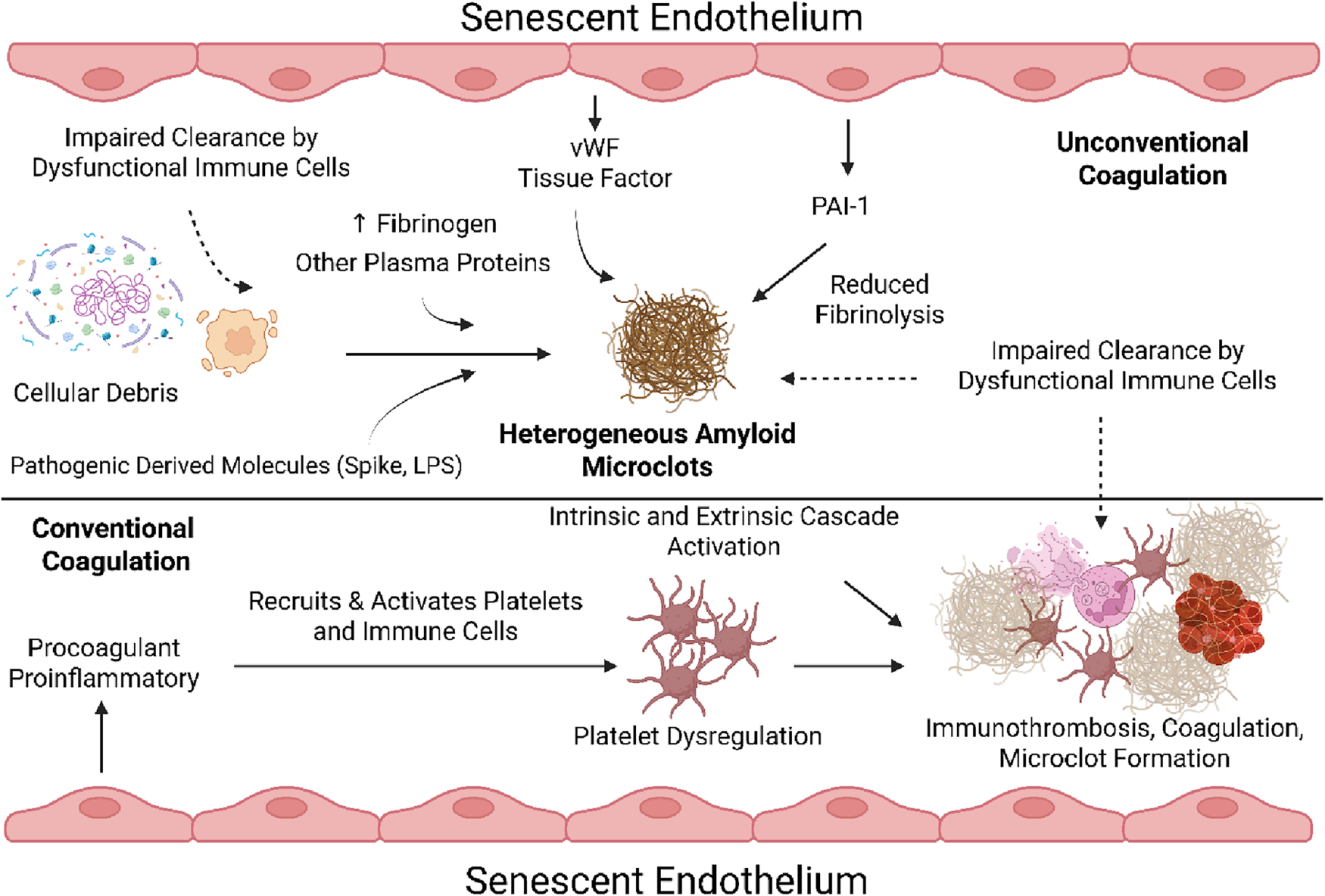
Procoagulant effects of the endothelial SASP. Senescent endothelial cells adopt a procoagulant and proinflammatory phenotype, leading to platelet dysregulation, immunothrombosis, and heterogeneous fibrinaloid microclot formation. The inability of dysfunctional immune cells to efficiently clear accumulating cellular debris and fibrin in circulation provides ample scaffold upon which coagulation can occur leading to microclot formation and persistence. This persistence is further exacerbated by elevations in PAI-1, which hinders fibrinolysis. (Created with the paid version of Biorender.com).

It is unknown if dysfunctional and/or senescent endothelial cells (and possibly the extracellular vesicles that they secrete [208]) are the primary inducers of heterogeneous fibrinaloid microclots. Microclots are heterogeneous aggregations in blood that stain positive with thioflavin T, an amyloid dye, and are composed of fibrin(ogen) and other plasma and intracellular proteins [110, 209]. It is hypothesized that extracellular vesicles, apoptotic bodies, and cellular debris can also induce microclot formation [210, 211]; if this is true, the increased load of heterogeneous microclots in long COVID might be explained by immune deficits which hinder clearance of cell debris and related particles in circulation. Additionally, these immune deficits can also explain the persistence of heterogeneous microclots, as leukocytes (as well as proteolytic mechanisms) are to be responsible for microclot clearance (Fig. 5). Senescent cells have been associated with amyloid pathology [212], and amyloid aggregates are capable of inducing cellular senescence [213]. While it has been shown that microclot induction can occur in the presence of bacterial inflammagens and SARS-CoV-2 spike protein [196–199, 214], other in vivo mechanisms—such as (senescent) endothelial-derived vesicle induction of platelet aggregation and subsequent anomalous clotting processes [215], activation of neutrophils and NETosis, accumulation of cellular debris and extracellular vesicles due to impaired immune function, and other potential mechanisms which will not be discussed here—need to be further investigated to explain the induction and persistence of these heterogeneous microclots. Since the endothelium plays a pivotal role in regulating coagulation, these links can guide future experiments investigating the induction of and the various phenotypes representative of the total population of heterogeneous microclots.

### Senescent endothelial cells and the relation to herpesviruses

There is a substantial amount of evidence implicating the herpesviridae EBV and HHV-6 in ME/CFS [13, 150, 216] and long COVID [217, 218]. Previously, we proposed that herpesviruses, specifically EBV and HHV-6, can infect and cause dysfunction in endothelial cells, causing symptoms associated with ME/CFS and long COVID [46].

Here, induction of senescence as a result of herpesvirus infection can occur in non-immune cells [219] and is specifically proposed to occur in endothelial cells (and also via indirect mechanisms). This would then lead to subsequent viral latency and cellular dysfunction that can contribute to pathophysiological mechanisms and symptoms, especially when clearance of these cells is implicated by a dysfunctional immune system and immune evasion strategies of viruses. We also recognize the importance of host susceptibility to such a pathological cycle induced by acute viral infection, but this is normally not known.

Herpesviridae are well known to be able to lie latent or dormant for many decades. Thus, as well as direct infection, immune alterations and endothelial senescence induced by the acute infection that initiated PVD may lead to herpesvirus reactivation. This may also be true for other infections and overall immunity.

### Future considerations

**Fig. 6:**
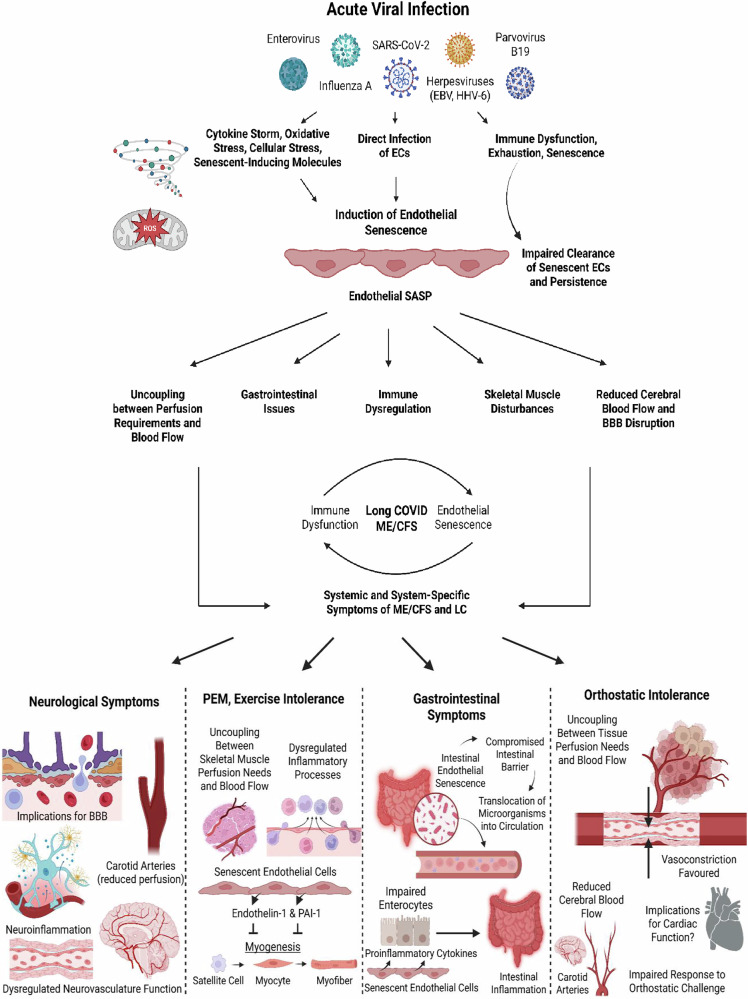
Summary of disease induction and maintenance with a focus on endothelial senescence, as well as its relation to symptom manifestation in long COVID and ME/CFS. (Created with the paid version of Biorender.com).

Confirming the details of the mechanisms proposed here (Fig. 6) in vivo will require the detection and quantification of cell-specific senescence (e.g., endothelial senescence) in patients, and these are still being developed [220]. There are currently no established biomarkers that are specific to virus-induced endothelial senescence, and the measurement of senescence-associated biomarkers in biological samples (e.g., urine, plasma, etc.) is likely insufficient to corroborate—specifically—endothelial senescence as a cause and driving factor of ME/CFS and long COVID; although, as discussed, many plasma markers in patients are suggestive of a senescent endothelium. Alternatively, the assessment of microRNAs expressed predominantly by endothelial cells, such as microRNA-126, can potentially provide insight into endothelial status and senescence [98].

What might offer valuable information are biopsies of both small and large vessels and subsequent immunohistochemistry and imaging processing of tissues. A study by Veroutis et al. [221] performed temporal artery biopsies and subsequently identified and quantified endothelial cell senescence in vascular tissue, establishing a senescence specific signature that can be used to study endothelial senescence in a neurological context [221]. A similar approach can be implemented to investigate the hypothesis of endothelial senescence as a cause and driving factor for neurological pathology and symptom manifestation. Advancements in senescence-imaging of musculoskeletal tissue can inform the investigation of endothelial senescence in muscle tissue and its relation to fatigue, exercise intolerance, and PEM. Other system-specific investigations can be carried out in this manner, e.g., the gastrointestinal vasculature.

Senotherapeutics are a new class of drugs and natural products that consist of two classes: senomorphics and senolytics [222]. Senolytics selectively clear senescent cells, while senomorphics are compounds that modulate their behaviour. If, as we argue here, endothelial senescence is central to ME/CFS and Long COVID, then it is reasonable that senotherapeutics and related treatments [220] might offer therapeutic benefit to ME/CFS and long COVID patients. Indeed, some have been shown to aid in COVID-19 disease severity and ameliorate neuropathology associated with SARS-CoV-2 infection [76, 77, 223]. Given the more general value of carefully chosen combinations of natural products in these diseases, it seems that a trawl among relevant natural products acting as senolytics or senomorphics might be worthwhile. In a similar vein, further studies are required to assess the burden of cellular senescence in ME/CFS and long COVID, and specifically the senescent burden of the endothelium and vasculature.

### Conclusion

ME/CFS and long COVID are the two most studied post-viral diseases, yet an official mechanistic explanation of disease induction and maintenance is lacking. Here, we offer a detailed mechanistic explanation involving the endothelium, immune system, and senescence. We propose that acute viral infection can result in direct and indirect modulation of endothelial cells that can lead to endothelial dysfunction and, in particular, that this dysfunction is represented by senescence. Supporting this theory is the known ability of SARS-CoV-2 to cause endothelial senescence. Immune deficits are prominent characteristics of ME/CFS and long COVID pathology, with notable deficits in leukocyte and complement function; furthermore, endothelial senescence can itself give rise to immune deficits, leading to amplification. We propose that these deficits sustain endothelial senescence by ineffectively clearing senescent endothelial cells. This, along with the expression of HLA-E by senescent endothelial cells and the consequential evasion of the immune system, can explain the chronicity of ME/CFS and long COVID pathology.

We suggest that this systems analysis can effectively link immune dysfunction with the continuing symptoms associated with ME/CFS and long COVID, and can explain the multisystemic nature of these conditions. Importantly, the hypothesis presented here might be extended to include the senescence of other vascular cells, such as smooth muscle cells, endothelial progenitor cells, and leukocytes. However, endothelial cells are here proposed to be the most significant cell type implicated due to their important physiological roles and capability to cause local and systemic pathology that can result in symptom manifestation (Fig. 6). On this basis, senotherapeutics would be seen to have the potential to provide a novel and valuable treatment strategy. Additionally, the idea of endothelial senescence as the primary point of pathology in ME/CFS and long COVID can account for why previous clinical trials without the implementation of senotherapeutics and related drugs failed to bring about significant amelioration.
